# Displacement between anterior chamber width obtained by swept-source anterior segment optical coherence tomography and white-to-white distance

**DOI:** 10.1371/journal.pone.0251990

**Published:** 2021-05-20

**Authors:** Teerajet Taechameekietichai, Anwell Nguyen, Sunee Chansangpetch, Shan C. Lin

**Affiliations:** 1 Faculty of Medicine, Chulalongkorn University, Bangkok, Thailand; 2 Department of Ophthalmology, University of California, San Francisco, San Francisco, California, United States of America; 3 Glaucoma Research Unit, Faculty of Medicine, Chulalongkorn University and King Chulalongkorn Memorial Hospital, Thai Red Cross Society, Bangkok, Thailand; 4 Glaucoma Center of San Francisco, San Francisco, California, United States of America; Saarland University, GERMANY

## Abstract

**Purpose:**

To determine the relationship between the external limbal location, represented by white-to-white (WTW) distance, and the actual angle location, represented by spur-to-spur (STS) and angle-to-angle (ATA) distances.

**Methods:**

166 eyes from 166 participants were imaged using CASIA2 anterior chamber optical coherence tomography (AS-OCT) and LenStar LS 900 optical biometer. The horizontal ATA and STS were measured using the swept-source Fourier-domain AS-OCT (CASIA2). The horizontal WTW was automatically measured using LenStar. The displacement lengths (DL) between WTW-STS and WTW-ATA were calculated. Bland-Altman plots and intraclass correlation were performed.

**Results:**

The study showed that WTW has a positive correlation with STS (ICC = 0.82, p<0.001) and ATA (ICC = 0.82, p<0.001). The Bland-Altman analysis demonstrated that the mean difference of WTW-STS is 0.10 mm (95% CI 0.06 to 0.14 mm) with limits of agreement of -0.42 to 0.63 mm between WTW and STS, and the mean difference of WTW-ATA is 0.10 mm (95% CI 0.06 to 0.15 mm) with limits of agreement of -0.48 to 0.64 mm between WTW and ATA. Linear regression with adjustment showed that a WTW value greater than 12.07 mm is associated with a greater DL (WTW-STS DL ß 0.18, p = 0.003; WTW-ATA DL ß 0.14, p = 0.03).

**Conclusions:**

Greater WTW was significantly associated with higher displacement of WTW from the two distances representing anterior chamber width. External limbal location may not accurately represent the actual angle location in eyes with larger WTW.

## Introduction

The external limbal location has served as a surrogate for the internal anterior chamber angle. The limbus is an area where the cornea transitions to the sclera [1]. Surgically, the anterior limbal line can be seen externally as the ridge where the conjunctiva inserts into the corneal margin. The posterior border of the blue-grey zone of the surgical limbus corresponds to Schwalbe’s line, which is directly adjacent to the anterior trabecular meshwork (TM) [2]. A white-to-white (WTW) distance, defined as an external measurement of the horizontal distance from limbus to limbus, is frequently used to estimate the anterior chamber (AC) width.

In the clinical setting, the WTW value has been integrated into several ophthalmic practices including calculation of the power of intraocular lens (IOL) implants [3–6], decisions on IOL width in angle-supported anterior chamber IOL (ACIOL) placement [7], and selection of the dimensions of a capsular tension ring [8].

Traditionally, the WTW distance is used to estimate the width of an angle to help decide the haptic sizing for an angle-supported ACIOL. The ACIOL footplates should be placed on the relatively inert scleral spur [7]. If the size of the ACIOL is too large, the lens could compress and damage the structures within the angle recess including the iris root and TM [9]. However, an underestimated ACIOL size may cause the lens to be mobile and injure the corneal endothelium [10, 11]. It has been suggested by many studies that the spur-to-spur (STS) distance can be approximated by adding a constant of 0.5 or 1 mm to the WTW value [12–16].

Other ocular procedures also utilize the limbus as an anatomic reference position for the scleral spur or the angle. Examples include the positioning of a scleral-fixated posterior chamber IOL and the entry point for the vitrector, infusion port, and light source in pars plana vitrectomy. Recently, a new technique called direct selective laser trabeculoplasty (SLT) or trans-scleral SLT has been proposed which acts by transmitting laser energy to the scleral tissue immediately anterior to the TM. Using the perilimbal sclera as a landmark for the TM, this new technique has demonstrated a promising reduction of intraocular pressure (IOP) in glaucoma patients [17].

However, the external limbal anatomy may not always accurately represent the angle and TM. The introduction of anterior segment optical coherence tomography (AS-OCT) devices allows an actual assessment of angle structures including the angle recess and scleral spur. Although TM is not consistently discernible with the currently available AS-OCT machines, its posterior border, the scleral spur, is visible and can be practically used as a reference structure for the TM [18]. With the use of AS-OCT, an actual AC width can be measured as the STS distance, a measurement from one scleral spur to the opposite scleral spur, and angle-to-angle (ATA) distance, the length of the line joining both angle recesses.

This study aims to evaluate the relationship between the external limbal location, represented by WTW distance, and the actual angle location, represented by STS and ATA distances.

## Materials and methods

The study adhered to the tenets of the Declaration of Helsinki for the use of human participants in research and was approved by the Institutional Review Board of the University of California, San Francisco. (IRB number 14–14328) The participants were recruited from September 2016 until June 2017, and all participants had provided signed informed consent prior to the research.

### Subjects

For this study, all individuals were enrolled from general ophthalmology and glaucoma clinics at the University of California, San Francisco. To be included, participants had to be 20 years or older and have no previous intraocular surgery or laser procedures. Moreover, participants who had any corneal or conjunctival abnormalities impeding proper evaluation of the AC by AS-OCT, active ocular infection, or inability to perform the test were excluded. If both eyes were eligible, one eye was randomly selected for the study.

The same experienced examiner was responsible for slit-lamp examination, gonioscopy, and Goldmann applanation tonometry for all participants. Shaffer angle grading of less than 2 for 180 degrees or greater was deemed as closed-angle, and eyes not meeting this criterion and without peripheral anterior synechiae were regarded as open-angle.

All the participants underwent LenStar and CASIA2 scans during the same visit. It is worth noting that all the scans were also done by a single experienced technician (AN). To prevent illumination-dependent or time-differences in ocular anatomy, the sequence of the devices used was selected at random.

### LenStar image acquisition

LenStar (LenStar LS-900, Haag-Streit AG, Koniz, Switzerland) scans were taken following the manufacturer’s instructions. Scans were all completed in a dimly lighted room without any mydriatics. All participants placed their forehead and chin on the headrest and chinrest, respectively. It was ensured that the side mark on the headrest bar lined up with the lateral canthus. During the scans, the participants kept both of their eyes open and quickly blinked when asked; the measurements were taken right after each blinking. Moreover, participants stayed positioned throughout the repeated measurements.

Using the installed proprietary software (version 2.12), the LenStar automatically calculated the WTW distance by placing the most appropriate circle on the edge of the iris, in order to approximate the best fit to the horizontal limbus [19]. Additionally, to make sure that the measurement was taken on the visual axis, the participants were required to fixate on an internal fixation light. Following the correction of the participant’s position, five scans were done in succession and the average result was obtained for the WTW distance. Throughout the acquisition process, the operator assessed that the scans were all in focus and centrally aligned for all participants.

### CASIA2 image acquisition

For CASIA2 (Tomey Corporation, Nagoya, Japan) acquisition, the angle analysis mode was used. This mode comprised 16 consecutive meridional scans (800 A-scans per line). The scan was carried out using the auto-alignment function.

CASIA2 was done following five minutes of dark adaptation (<1 lux illumination at the imaging plane). Additionally, the participants’ eyelids were held open gently against the orbital rim, to avoid pressure to the globe, by an assistant. A live preview was also used to check for any angle deformation in real-time. A full 360-degree angle was inspected and if deformation was noted a subsequent repeat imaging was done up to three times. The subject was excluded if three of the repeats still demonstrate deformation of the angle. Throughout the acquisition process, the operator assessed each image for its quality. This included checking for clear visualization of the scleral spur and angle recess area and ensuring that there is no shadow or motion artifact. For the analysis, only the images with horizontal (180-degree) alignment were used, and any horizontal image with poor visualisation of the scleral spurs and angle areas were excluded.

### Image analysis

For each participant, only the qualifying image from each machine was used for analysis. Only one fellowship-trained grader (SC) was responsible for all of the image analysis. The grader was blinded to the clinical and gonioscopic findings. The image analysis for each machine was done separately and the image order was selected randomly to avoid potential association with images with other devices.

LenStar is an optical biometer that is able to evaluate different ocular parameters in a single procedure. It utilizes a 950-nm LED and high-resolution color photography to automatically measure the WTW distance [20]. The STS and ATA distances were obtained by a built-in 2D Analysis program in CASIA2 which allows the measurements, structural outlines, and reference lines to be automatically calculated. While the CASIA2 is pre-programmed for 2D Analysis to delineate the temporal and nasal scleral spurs, to ensure the correct grading a single grader (SC) manually reviewed and re-adjusted the scleral spur marks if indicated. Similarly, to ensure precision, the grader also reviewed the automatically produced reference lines and image outlines from the program and if required the outline tracer was adjusted.

The following criteria were used to define scleral spur position: (1) the interface line between the less reflective ciliary muscle and sclera intersects with the inner corneal margin [21], (2) where there is an alteration to the curvature in the corneoscleral-aqueous interface [22], and (3) the apex of an internal projection of the inner margin of the cornea and TM.

**Fig 1:**
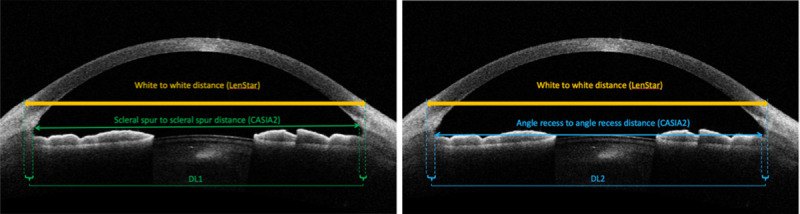
Image of anterior chamber in horizontal meridian using CASIA2 anterior segment optical coherence tomography. DL1 is the displacement lengths of white-to-white distance minus scleral spur-to-scleral spur distance. DL2 is the displacement lengths of white-to-white distance minus angle-to-angle distance.

The displacement lengths (DL) were then calculated using WTW distance minus STS distance for the WTW-STS DL distance, and WTW distance minus ATA distance for the WTW-ATA DL (Fig 1).

### Statistical analysis

All the measurements are shown as the mean and standard deviation. Intraclass correlation coefficients (ICC) for two-way mixed-effects model and Bland-Altman plots with trend were used to access the agreement between WTW versus STS distance, and WTW versus ATA distance. The WTW was then divided into quartiles and treated as predictors in a multivariable linear regression for both DL outcomes with an adjustment for possible confounding variable including age, gender, angle status and axial length. Statistical analysis was performed using Stata (Stata/MP 13.0, College Station, TX). A P value of less than 0.05 was considered statistically significant.

## Results

**Fig 2:**
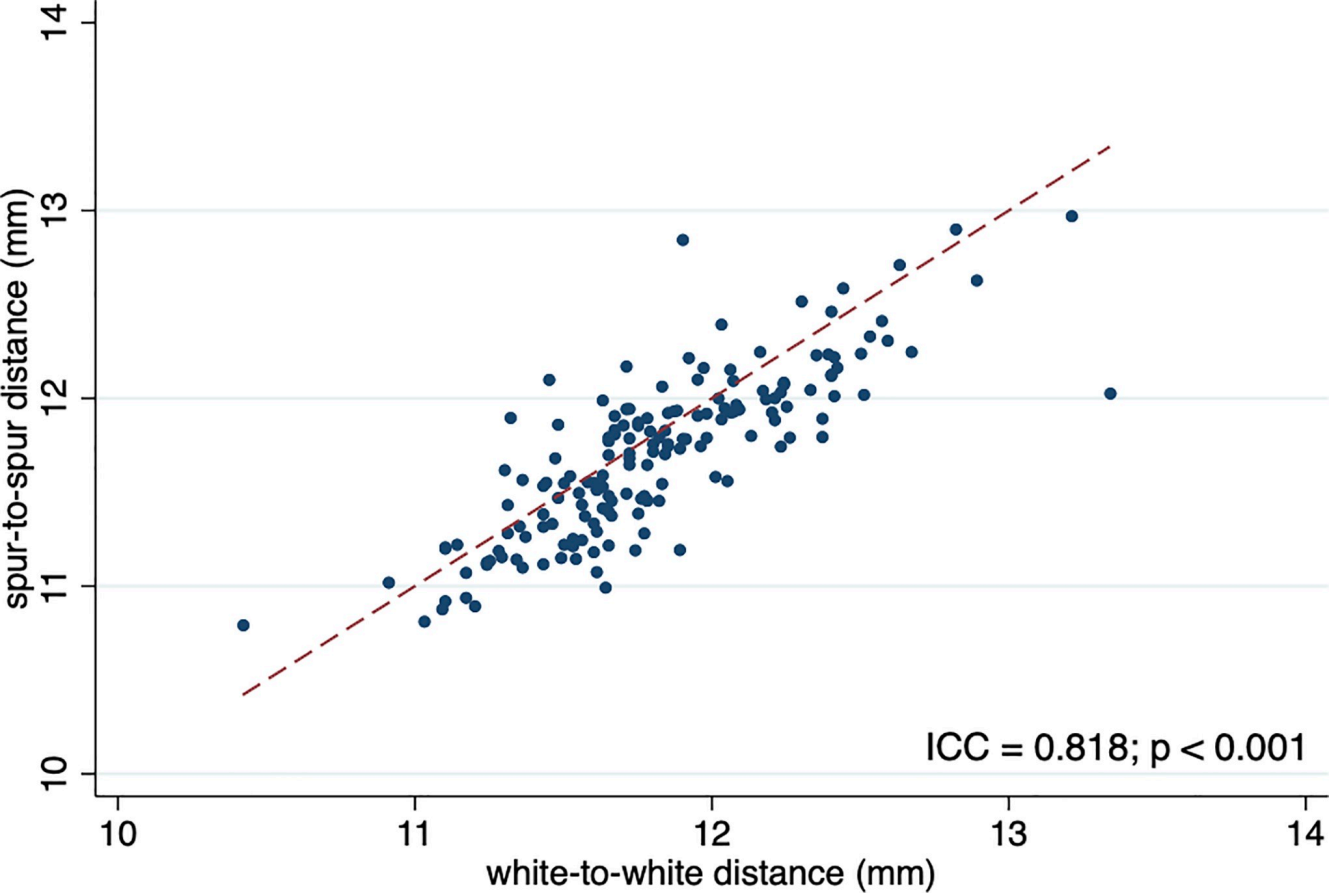
Scatter plot of spur-to-spur distance with respect to white-to-white distance. ICC, intraclass correlation coefficient; dashed line indicates the equality line.

**Fig 3:**
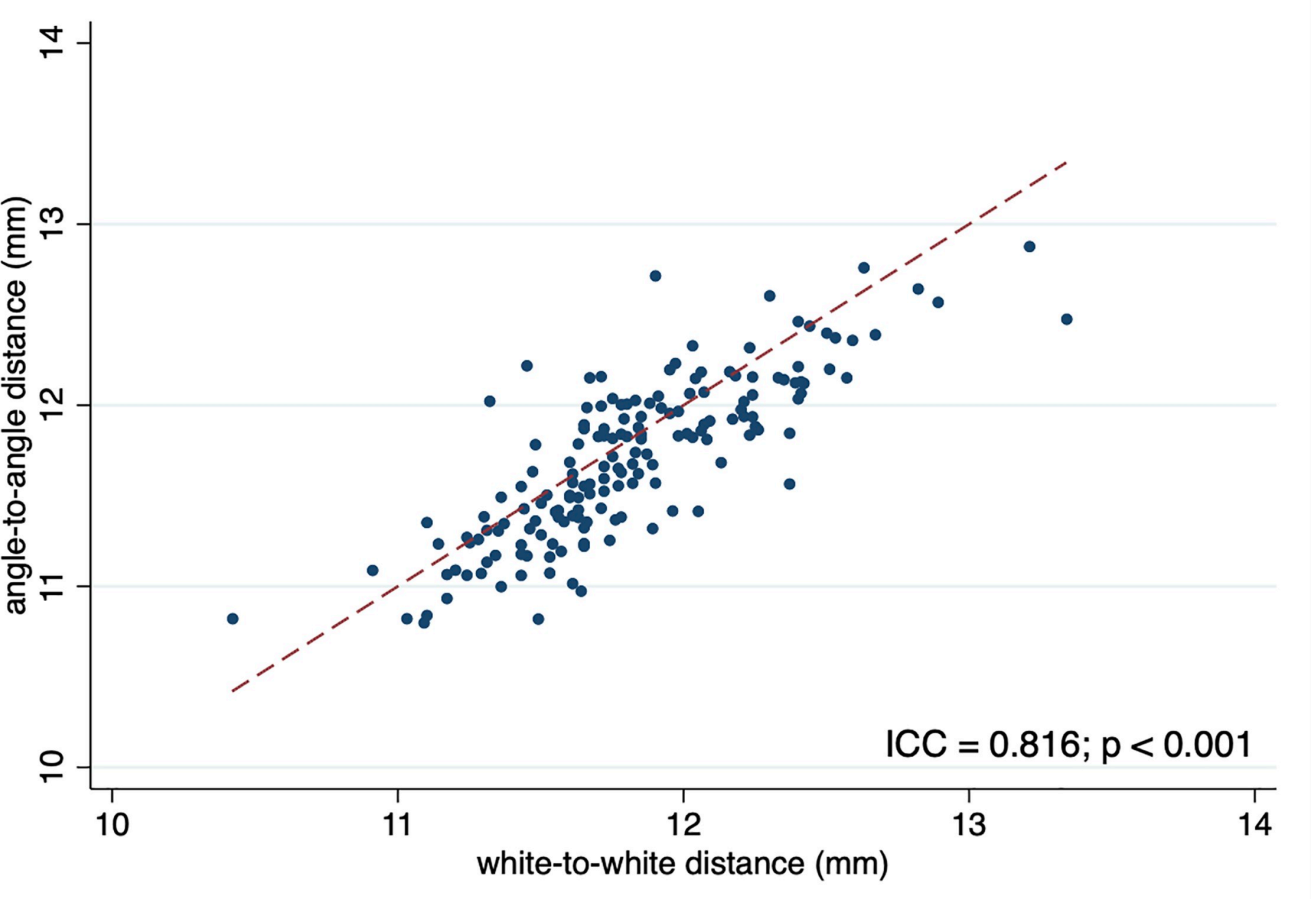
Scatter plot of angle-to-angle distance with respect to white-to-white distance. ICC, intraclass correlation coefficient; dashed line indicates the equality line.

This study included 166 eyes of 166 participants. From this cohort, we were able to measure three separate but related values for each subject. The mean age was 66.5 years (range 41–93, SD 10.5). This population was comprised of 67 males (40%) and 99 females (60%). Table 1 shows the clinical characteristics of the participants. The average (SD) of WTW, STS, and ATA were 11.8 (0.4), 11.7 (0.4), and 11.7 (0.4), respectively. WTW showed significant positive correlation with STS (ICC = 0.82, p<0.001, 95% CI 0.76 to 0.86) and ATA (ICC = 0.82, p<0.001, 95% CI 0.76 to 0.86) (Figs 2 and 3).

**Table 1:** Clinical characteristics.

Characteristics	N = 166
Age (year)	66.5 (10.5)
Gender	
• Male	67 (40%)
• Female	99 (60%)
Ethnic Group	
• Caucasian	54 (32%)
• Asian	81 (49%)
• African American	21 (13%)
• Hispanic	8 (5%)
• Native American	2 (1%)
Angle status	
• Open	119 (72%)
• Closed	47 (28%)
Laterality	
• Right	90 (54%)
• Left	76 (46%)
Axial length (mm)	24.15 (2.0)

Mean (SD) or n (%).

**Fig 4:**
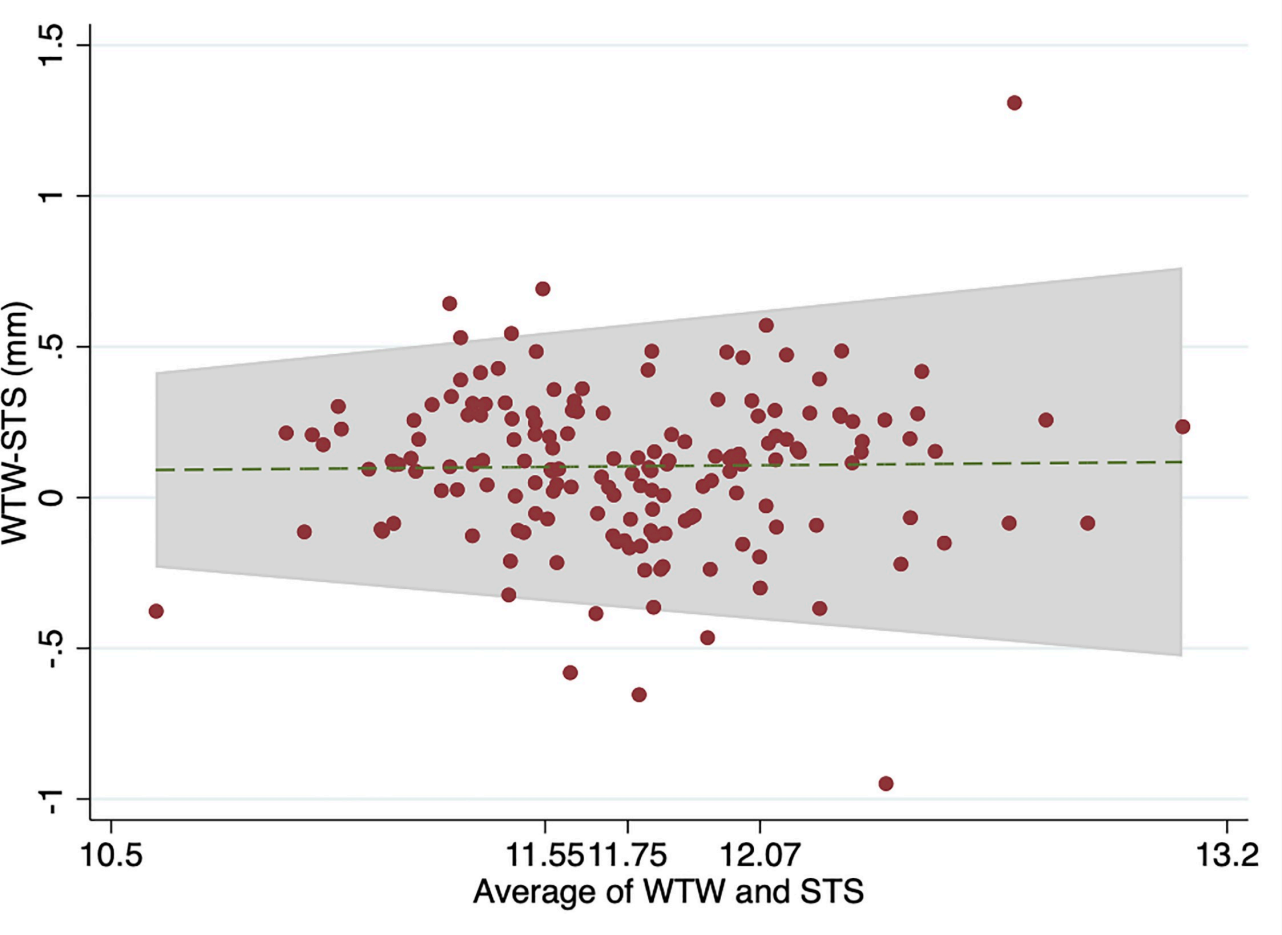
Bland-Altman plots with trend for WTW-STS and average of WTW and STS. WTW value of equal to or less than 11.55 mm is a first quartile, value of 11.55 to 11.75 mm represents the second quartile, value of 11.76 to 12.07 mm represents the third quartile, and value of greater than 12.07 mm is a fourth quartile. WTW white-to-white distance; STS spur-to-spur distance.

**Fig 5:**
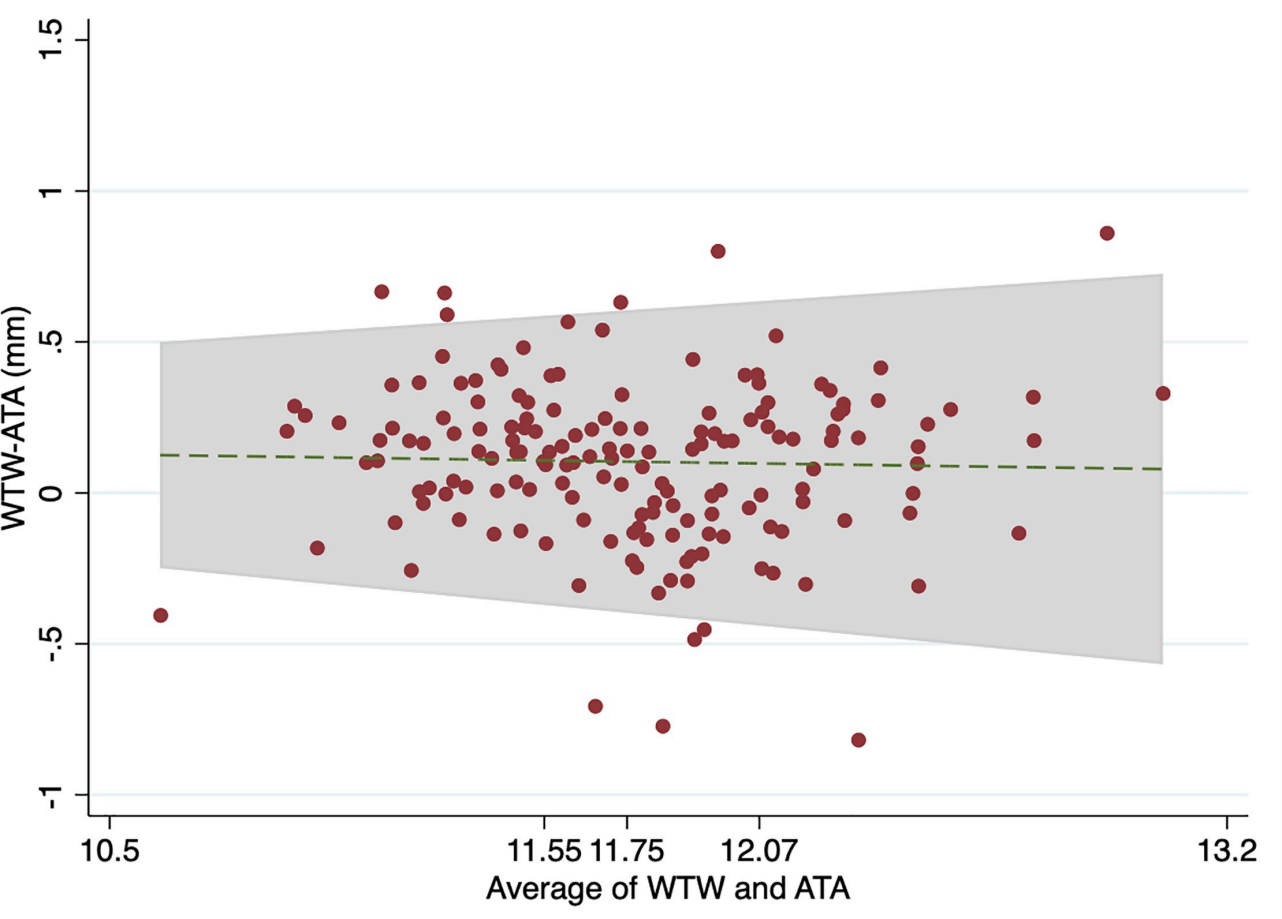
Bland-Altman plots with trend for WTW-ATA and average of WTW and ATA. WTW value of equal to or less than 11.55 mm is a first quartile, value of 11.55 to 11.75 mm represents the second quartile, value of 11.76 to 12.07 mm represents the third quartile, and value of greater than 12.07 mm is a fourth quartile. WTW white-to-white distance; ATA angle-to-angle distance.

The Bland-Altman analysis revealed a mean difference (WTW-STS) of 0.10 mm (95% CI 0.06 to 0.14 mm) with limits of agreement (LOAs) of -0.42 to 0.63 mm between WTW and STS, and a mean difference (WTW-ATA) of 0.10 mm (95% CI 0.06 to 0.15 mm) with LOAsof -0.48 to 0.64 mm between WTW and ATA. Figs 4 and 5 showed Bland-Altman plots adjusted for trend. The plots revealed that the DLs became wider as the measurement values increased. A total of 156/166 (94%) had the WTW-ATA DL and 160/166 (96%) WTW-STS DL of less than 0.5 mm.

The WTW distance was divided into four categories by quartile. The DLs of each category are shown in Table 2. Linear regression with adjustment for age, gender, angle status and axial length showed that the 4th quartile WTW (WTW > 12.07 mm) was significantly associated with greater DLs (WTW-STS DL ß 0.175, p = 0.003; WTW-ATA DL ß 0.139, p = 0.028).

**Table 2:** Displacement length for the four categories by white-to-white distance in quartile.

Displacement length	Mean	SD	ß coeff.	P value	95% CI
WTW-STS (mm)	0.10	0.26			
▪ 1st quartile WTW < 11.55 mm	0.02	0.24	reference	reference	reference
▪ 2nd quartile WTW 11.55–11.75 mm	0.08	0.25	0.03	0.586	-0.08 to 0.14
▪ 3rd quartile WTW 11.76–12.07 mm	0.08	0.27	0.08	0.176	-0.03 to 0.19
▪ 4th quartile WTW > 12.07 mm	0.24	0.25	0.18	0.003	0.06 to 0.29
				P for trend <0.001	
WTW-ATA (mm)	0.10	0.27			
▪ 1st quartile WTW < 11.55 mm	0.05	0.28	reference	reference	reference
▪ 2nd quartile WTW 11.55–11.75 mm	0.08	0.27	-0.002	0.996	-0.12 to 0.12
▪ 3rd quartile WTW 11.76–12.07 mm	0.05	0.26	0.02	0.789	-0.11 to 0.14
▪ 4th quartile WTW > 12.07 mm	0.24	0.22	0.14	0.028	0.02 to 0.26
				P for trend 0.008	

Adjustment for age, gender, angle status, and axial length; CI, confidence interval.

## Discussion

This study analyzed the relationship between the WTW distance and the location of the AC angle, which was represented by STS and ATA. The result demonstrated a significant correlation between WTW and the STS and ATA distances. Additionally, the results suggested that the WTW distance with a greater value has a larger dissociation between WTW and STS. This is especially true for the WTW distance within the 4th quartile, where the WTW is greater than 12.07 mm.

Our results are in agreement with many studies that have also shown significant correlations between WTW, STS and ATA distance in a horizontal meridian [23–25]. In contrast, in a study conducted by Pinero et al. [14], where the WTW was measured with corneal topography and ATA by an OCT, a poor correlation between ATA and WTW was found. However, these previous studies used different imaging techniques to evaluate the various parameters. It has been suggested that the difference in the studies that measured horizontal WTW could be because of the use of manual or automatic measurements [14]. It is intrinsically difficult to precisely determine the point where the cornea finishes, and the sclera begins.

The Bland–Altman plots between WTW–STS and WTW–ATA showed minor differences. The results suggest that the ACIOL size should be based on the WTW distance without adjustment rather than the conventional method of adding 0.5 to 1 mm to the WTW value. In line with our findings, the study from Bruner et al. reported that adding a small constant to the horizontal WTW distance could make the value of STS higher than it really is [7]. Moreover, the difference of less than 0.5 mm does not affect the ACIOL selection because the available sizes usually increase in steps of 0.5 mm [7]. Our study found that 96% of the subjects had a DL of less than 0.5 mm. It should be noted that our results may only be applicable to the horizontal ACIOL placement.

In cataract surgery, the accuracy of IOL calculation theoretically depends on the precision of predictive effective lens position which is currently estimated by IOL formulas utilizing several biometric parameters including WTW. For example, WTW is one of the parameters that the Holladay 2 formula uses to estimate postoperative anterior chamber depth [3]. However, our findings suggested that the internal ocular dimension was not always accurately represented by WTW as the WTW-STS and WTW-ATA displacements became wider in the larger WTW eyes. The role of STS and ATA in comparison to WTW in IOL calculation warrants further studies.

An investigational direct SLT utilizes external limbal location as a landmark for the laser application. This new technique offers considerable clinical potential. Not only does it provide the possible benefits of conventional SLT in terms of IOP reduction and cost-effectiveness [26], but it also possesses other advantages such as being a non-contact procedure. A study by Geffen et al. [17], which compared the effectiveness of direct SLT against the standard SLT, demonstrated that the average IOP reduction after 6 months in the study and control groups were 23.4% and 27.1% respectively. After a year of follow-up, the study concluded that a new direct SLT could be as effective as conventional SLT treatment [17]. The recent global pandemic has caused concerns regarding the transmission of infection during procedures. As direct SLT is a contactless procedure, this technique could help to ameliorate the risk of infection being transmitted between the patients. Since the visualisation of the angle is not needed in direct SLT, it also has the potential to be used in narrow-angle eyes as well [27]. The ability to more precisely target and localize the structures for treatment—in this case, the TM—even using external landmarks such as the limbus can perhaps further enhance the effectiveness of this novel delivery of SLT therapy.

Although overall, there were positive correlations between the WTW and AC widths; the use of WTW distance to locate TM would demand a greater precision. This new direct SLT technique applies the laser over the external limbal border because it was approximated that the TM is located underneath the border [17]. Our study supports the use of this laser landmark in eyes with small WTW value (< 11.55 mm) because we found that only a small displacement of 0.02 mm occurs between WTW and STS, which equates to 0.01 mm on each side of the angle. Studies have shown that the TM is located anterior to the scleral spur and has approximately 500 to 700 μm in length [28, 29]. This makes an external limbal location an acceptable landmark for a laser spot with 0.4-mm in diameter to be delivered to the TM in patients with a small WTW value. However, our results indicate that this may not always be true in patients with a higher WTW value, especially when the WTW is greater than 12.07 mm. In normal-to-large WTW patients, because of the greater WTW-STS DL, the laser spot may not be placed right above the TM, but instead only the posterior part of the TM. For example, the average WTW-STS DL in eyes with WTW > 12.07 mm is 0.24 mm which equate to 0.12 mm on each side of the angle. Applying the 0.4-mm diameter laser over the external limbal border would approximately cover 0.08 mm of the posterior TM, which is only about one-tenth of the total TM length. This inaccurate estimation of the TM location could lead to SLT laser being applied only to a small area of TM which would deliver a less effective treatment.

One of the strengths of this study is that a single technician and image assessor were used to operate the machines and examine the images taken, respectively. This would help to reduce the variability of the image acquisition and the parameter being measured. Compared to the previous studies we also used a relatively newer device to measure different parameters, which may deliver more accurate findings. For instance, the CASIA2 has a higher resolution relative to the widely used previous model CASIA SS1000. Our study also separated patients into different quartiles so that it is more applicable to the clinical practice. We also have a greater number of subjects (166 eyes) than other previous studies (no more than 100 eyes).

This study has potential limitations. First of all, our study looked at the parameters only in the horizontal meridian; however, the relationship between limbal location and the angle structures are likely varied in different meridians. Future studies should be carried out to properly determine the correlation of WTW, STS and ATA in different axes. Secondly, we did not perform the precision test in the study. The value of DLs was small and could potentially be confounded by the devices’ variability. Nevertheless, prior studies reported good repeatability with small coefficients of variation of 0.82% for STS and 0.44% for ATA measured by CASIA2, and 0.10% for WTW measured by LenStar LS-900 [30–32]. In addition, Xu et al. reported the LOAs of STS in their repeatability test was -0.24 to 0.16 mm and Saito et al. found the LOAs of ATA was -0.16 to 0.18 mm [30, 32]. Our data found the mean DLs of both STS and ATA at the 4th quartile WTW were 0.24 mm. Using the same devices, the LOAs in previous reports were equal to or smaller than the magnitude of the statistically significant DLs in our study. Third, most of the subjects in our study have open-angle status, and there were 28% who had closed-angle status. Another limitation of our study is that a large proportion of the patients were Asian (49%). Comparing with the Montés-Micó et al. study [25], our mean horizontal STS values of 11.7 mm are slightly smaller than their results (STS of 11.9 mm). However, all of the subjects in the Montés-Micó et al. study were female Caucasians [25]. As Asian eyes are found to have smaller STS than Caucasians, the difference in ethnicity in our sample may have influenced the results [33]. Considering this limitation, we feel that to gain a better understanding of the correlation between the distances it would be important for further studies with a bigger sample size and different ethnic groups to be carried out.

In conclusion, the WTW distance has a positive correlation with STS and ATA. The DLs became wider as the measurement values increased. Based on our data, we demonstrate that the actual horizontal WTW value could be used to determine the size of an ACIOL without having any adjustment. Our study also indicates that the perilimbal sclera may not be a good estimator of scleral spur location for direct SLT, since it may require a more precise laser application, especially for eyes with a larger WTW, for the greatest effectiveness.

## Supporting information

S1 Data (XLSX)

## References

[1] Le Q, Chen Y, Yang Y, Xu J. Measurement of corneal and limbal epithelial thickness by anterior segment optical coherence tomography and in vivo confocal microscopy. BMC Ophthalmol. 2016;16(1):163. Epub 2016/09/21. 10.1186/s12886-016-0342-x 27645227 PMC5029042

[2] Ahmed E. Comprehensive Manual of Ophthalmology: Jaypee Brothers,Medical Publishers Pvt. Limited; 2010.

[3] Hoffer KJ. Clinical results using the Holladay 2 intraocular lens power formula. J Cataract Refract Surg. 2000;26(8):1233–7. Epub 2000/09/29. 10.1016/s0886-3350(00)00376-x . 11008054

[4] Fernandez J, Rodriguez-Vallejo M, Martinez J, Tauste A, Hueso E, Pinero DP. Confounding sizing in posterior chamber phakic lens selection due to white-to-white measurement bias. Indian J Ophthalmol. 2019;67(3):344–9. Epub 2019/02/20. 10.4103/ijo.IJO_613_18 30777951 PMC6407395

[5] Salouti R, Nowroozzadeh MH, Zamani M, Ghoreyshi M, Salouti R. Comparison of horizontal corneal diameter measurements using Galilei, EyeSys and Orbscan II systems. Clin Exp Optom. 2009;92(5):429–33. Epub 2009/08/18. 10.1111/j.1444-0938.2009.00407.x . 19681922

[6] Venkataraman A, Mardi SK, Pillai S. Comparison of Eyemetrics and Orbscan automated method to determine horizontal corneal diameter. Indian J Ophthalmol. 2010;58(3):219–22. Epub 2010/04/24. 10.4103/0301-4738.62647 20413925 PMC2886253

[7] Bruner C, Skanchy DF, Wooten JP, Chuang AZ, Kim G. Anterior chamber lens sizing: Comparison of white-to-white and scleral spur-to-scleral spur methods. J Cataract Refract Surg. 2020;46(1):95–101. Epub 2020/02/13. 10.1016/j.jcrs.2019.08.043 . 32050238

[8] Weber CH, Cionni RJ. All about capsular tension rings. Curr Opin Ophthalmol. 2015;26(1):10–5. Epub 2014/11/13. 10.1097/ICU.0000000000000118 . 25390861

[9] Apple DJ, Brems RN, Park RB, Norman DK, Hansen SO, Tetz MR, et al. Anterior chamber lenses. Part I: Complications and pathology and a review of designs. J Cataract Refract Surg. 1987;13(2):157–74. Epub 1987/03/01. 10.1016/s0886-3350(87)80131-1 . 3572772

[10] Dajee KP, Abbey AM, Williams GA. Management of dislocated intraocular lenses in eyes with insufficient capsular support. Curr Opin Ophthalmol. 2016;27(3):191–5. Epub 2016/02/26. 10.1097/ICU.0000000000000260 . 26913739

[11] Ravalico G, Botteri E, Baccara F. Long-term endothelial changes after implantation of anterior chamber intraocular lenses in cataract surgery. J Cataract Refract Surg. 2003;29(10):1918–23. Epub 2003/11/08. 10.1016/s0886-3350(02)02052-7 . 14604711

[12] Heslin KB. Is "white-to-white" right? J Am Intraocul Implant Soc. 1979;5(1):50–1. Epub 1979/01/01. 10.1016/s0146-2776(79)80036-1 . 438077

[13] Karickhoff JR. Instruments and techniques for anterior chamber implants. Arch Ophthalmol. 1980;98(7):1265–7. Epub 1980/07/01. 10.1001/archopht.1980.01020040117018 . 7396781

[14] Piñero DP, Plaza Puche AB, Alió JL. Corneal diameter measurements by corneal topography and angle-to-angle measurements by optical coherence tomography: evaluation of equivalence. J Cataract Refract Surg. 2008;34(1):126–31. Epub 2008/01/01. 10.1016/j.jcrs.2007.10.010 . 18165092

[15] Roberts JC. A method for anterior chamber lens size determination. J Am Intraocul Implant Soc. 1981;7(2):171. Epub 1981/04/01. 10.1016/s0146-2776(81)80080-8 . 7263492

[16] Werner L, Izak AM, Pandey SK, Apple DJ, Trivedi RH, Schmidbauer JM. Correlation between different measurements within the eye relative to phakic intraocular lens implantation. J Cataract Refract Surg. 2004;30(9):1982–8. Epub 2004/09/03. 10.1016/j.jcrs.2003.10.041 . 15342066

[17] Geffen N, Ofir S, Belkin A, Segev F, Barkana Y, Kaplan Messas A, et al. Transscleral Selective Laser Trabeculoplasty Without a Gonioscopy Lens. J Glaucoma. 2017;26(3):201–7. Epub 2016/09/17. 10.1097/IJG.0000000000000464 . 27636593

[18] Tun TA, Baskaran M, Zheng C, Sakata LM, Perera SA, Chan AS, et al. Assessment of trabecular meshwork width using swept source optical coherence tomography. Graefes Arch Clin Exp Ophthalmol. 2013;251(6):1587–92. Epub 2013/02/26. 10.1007/s00417-013-2285-8 . 23436037

[19] Huang J, Savini G, Su B, Zhu R, Feng Y, Lin S, et al. Comparison of keratometry and white-to-white measurements obtained by Lenstar with those obtained by autokeratometry and corneal topography. Cont Lens Anterior Eye. 2015;38(5):363–7. Epub 2015/05/10. 10.1016/j.clae.2015.04.003 . 25956573

[20] Shin MC, Chung SY, Hwang HS, Han KE. Comparison of Two Optical Biometers. Optom Vis Sci. 2016;93(3):259–65. Epub 2016/01/14. 10.1097/OPX.0000000000000799 . 26760579

[21] Seager FE, Wang J, Arora KS, Quigley HA. The effect of scleral spur identification methods on structural measurements by anterior segment optical coherence tomography. J Glaucoma. 2014;23(1):e29–38. Epub 2013/06/29. 10.1097/IJG.0b013e31829e55ae . 23807354

[22] Sakata LM, Wong TT, Wong HT, Kumar RS, Htoon HM, Aung HT, et al. Comparison of Visante and slit-lamp anterior segment optical coherence tomography in imaging the anterior chamber angle. Eye (Lond). 2010;24(4):578–87. Epub 2009/06/13. 10.1038/eye.2009.141 . 19521435

[23] Petermeier K, Suesskind D, Altpeter E, Schatz A, Messias A, Gekeler F, et al. Sulcus anatomy and diameter in pseudophakic eyes and correlation with biometric data: evaluation with a 50 MHz ultrasound biomicroscope. J Cataract Refract Surg. 2012;38(6):986–91. Epub 2012/05/26. 10.1016/j.jcrs.2011.12.027 . 22624897

[24] Nemeth G, Hassan Z, Szalai E, Berta A, Modis L Jr. Comparative analysis of white-to-white and angle-to-angle distance measurements with partial coherence interferometry and optical coherence tomography. J Cataract Refract Surg. 2010;36(11):1862–6. Epub 2010/10/30. 10.1016/j.jcrs.2010.05.017 . 21029893

[25] Montés-Micó R, Tañá-Rivero P, Aguilar-Córcoles S, Ruiz-Santos M, Rodríguez-Carrillo MD, Ruiz-Mesa R. Angle-to-angle and spur-to-spur distance analysis with high-resolution optical coherence tomography. Eye Vis (Lond). 2020;7:42. Epub 2020/08/22. 10.1186/s40662-020-00208-0 32821763 PMC7429782

[26] Gazzard G, Konstantakopoulou E, Garway-Heath D, Garg A, Vickerstaff V, Hunter R, et al. Selective laser trabeculoplasty versus eye drops for first-line treatment of ocular hypertension and glaucoma (LiGHT): a multicentre randomised controlled trial. Lancet. 2019;393(10180):1505–16. Epub 2019/03/14. 10.1016/S0140-6736(18)32213-X 30862377 PMC6495367

[27] Garg A, Gazzard G. Selective laser trabeculoplasty: past, present, and future. Eye (Lond). 2018;32(5):863–76. Epub 2018/01/06. 10.1038/eye.2017.273 29303146 PMC5944654

[28] Kasuga T, Chen YC, Bloomer MM, Hirabayashi KE, Hiratsuka Y, Murakami A, et al. Trabecular meshwork length in men and women by histological assessment. Curr Eye Res. 2013;38(1):75–9. Epub 2012/06/30. 10.3109/02713683.2012.700757 . 22742780

[29] Fernandez-Vigo JI, Garcia-Feijoo J, Martinez-de-la-Casa JM, Garcia-Bella J, Fernandez-Vigo JA. Morphometry of the trabecular meshwork in vivo in a healthy population using fourier-domain optical coherence tomography. Invest Ophthalmol Vis Sci. 2015;56(3):1782–8. Epub 2015/02/24. 10.1167/iovs.14-16154 . 25698706

[30] Xu BY, Mai DD, Penteado RC, Saunders L, Weinreb RN. Reproducibility and Agreement of Anterior Segment Parameter Measurements Obtained Using the CASIA2 and Spectralis OCT2 Optical Coherence Tomography Devices. J Glaucoma. 2017;26(11):974–9. Epub 2017/09/21. 10.1097/IJG.0000000000000788 28930883 PMC5670007

[31] Shetty N, Kaweri L, Koshy A, Shetty R, Nuijts R, Roy AS. Repeatability of biometry measured by IOLMaster 700, Lenstar LS 900 and Anterion, and its impact on predicted intraocular lens power. J Cataract Refract Surg. 2020. Epub 2020/12/01. 10.1097/j.jcrs.0000000000000494 . 33252565

[32] Saito A, Kamiya K, Fujimura F, Takahashi M, Shoji N. Comparison of angle-to-angle distance using three devices in normal eyes. Eye (Lond). 2020;34(6):1116–20. Epub 2019/10/30. 10.1038/s41433-019-0653-2 31659288 PMC7253441

[33] Qin B, Tang M, Li Y, Zhang X, Chu R, Huang D. Anterior segment dimensions in Asian and Caucasian eyes measured by optical coherence tomography. Ophthalmic Surg Lasers Imaging. 2012;43(2):135–42. Epub 2012/02/11. 10.3928/15428877-20120102-03 22320411 PMC3402168

